# Myasthenia gravis exacerbation after melatonin administration: case series from a tertiary referral centre

**DOI:** 10.1186/s12883-020-01975-y

**Published:** 2020-11-04

**Authors:** Velina Nedkova-Hristova, Valentina Vélez-Santamaría, Carlos Casasnovas

**Affiliations:** 1 Neuromuscular Diseases Unit, Department of Neurology, Bellvitge University Hospital -IDIBELL, Carrer de la Feixa Llarga, s/n, 08907, L’Hospitalet de Llobregat, Barcelona, Spain; 2 Bellvitge Institute for Biomedical Research (IDIBELL), L’Hospitalet de Llobregat, Barcelona, Spain; 3 Multidisciplinary Unit of Familiar Amyloidosis, Bellvitge University Hospital-IDIBELL, Barcelona, Spain; 4 Special Group on Metabolic Diseases – IDIBELL, L’Hospitalet de Llobregat, Barcelona, Spain; 5 Network Centre for Biomedical Research on Rare Diseases (CIBERER), Carlos III Health Institute (Ministry of the Economy and Competitiveness), Madrid, Spain

**Keywords:** Melatonin, Myasthenia gravis, Corticosteroid, Immunosuppressant, Case report

## Abstract

**Background:**

Myasthenia gravis is an autoimmune disease mediated by antibodies against proteins associated with the postsynaptic membrane of the neuromuscular junction. Several drugs may trigger an exacerbation of the disease. Melatonin supplements are widely used for the treatment of insomnia as they are well tolerated with few side effects. The role of melatonin in the immune system and its effects in autoimmune disorders remain uncertain.

**Case presentation:**

We identified three patients in our referral centre from 2014 to 2019 who presented a worsening within days or weeks of starting melatonin. Two of them stopped the treatment without clinical improvement in the next week. Increasing dose of corticosteroids did not lead to clinical improvement in the next month and one of the patients was finally administered intravenous immunoglobulins.

**Conclusion:**

Melatonin may trigger exacerbations of myasthenia gravis, probably due to an upregulation of the adaptive immune system and an interaction with the corticosteroids and other immunosuppressant treatments. We consider that melatonin should be administered with caution in these patients.

## Background

Myasthenia gravis (MG) is an autoimmune disease mediated by antibodies against the acetylcholine receptor (anti-AchR), the muscle-specific kinase (anti-MuSK) or other proteins associated with the postsynaptic membrane of the neuromuscular junction. The main clinical manifestations are muscle weakness and fatigue (which worsens after repeated muscular effort) [1]. Symptoms may be exclusively ocular (ocular MG), with diplopia and/or ptosis, or generalized (generalized MG), involving weakness of the limb, trunk, dysphagia, dysphonia or respiratory insufficiency.

Several drugs, notably sedatives and some antibiotics may trigger an exacerbation of MG and hence they should be used with caution, weighing up the risks and benefits in each patient. Some over-the-counter pharmacy products may also contain substances that worsen the symptoms of MG.

Melatonin (N-acetyl-5-methoxytryptamine) is a hormone that is primarily released by the pineal gland and which is involved in regulating the sleep-wake cycle. Levels of melatonin decrease with age and this could be one of the reasons why older adults are more prone to suffer from insomnia [2].

Because exogenous melatonin binds to the same receptors as the endogenous form, it is effective as a treatment for insomnia [2–5]. Melatonin supplements are well tolerated, with few side effects (headache, dizziness, nausea and drowsiness) and no problems of tolerance. This is an important advantage over other sedative-hypnotic drugs such as antihistamines, antidepressants and benzodiazepines.

In addition to regulating the sleep-wake cycle, it is becoming increasingly clear that melatonin can also have immunomodulatory effects. There are now several reports describing the onset or exacerbation of autoimmune disorders after taking supplements containing melatonin or one of its analogues [6–9]. Here we report the first three cases of patients with a clinical exacerbation of MG after administration of melatonin, (Table 1).

**Table 1:** Clinical characteristics of the patients

Patient	Patient 1	Patient 2	Patient 3
Age (years)	49	87	35
Type of MG	Generalized	Generalized	Generalized
Antibodies	Double seronegative	Positive for anti-AchR	Positive for anti-AchR
Treatment	PDN 10 mg/48 h and MA 360 mg/12 h	PDN 10 mg/48 h	PDN 10 mg/48 h and MA 540 mg/24 h
MGFA prior to taking melatonin	0	I	I
MGFA-PIS prior to taking melatonin	PR	MM1	MM3
ADL score prior to taking melatonin	0	0	2
Melatonin dose	1.95 mg/day	1.9 mg/day	1.95 mg/day
Time from start of melatonin to worsening	3 weeks	2 days	7 days
Deterioration in ADL score	5	4	7
Melatonin ceased	Yes	No	Yes
Treatment received	Increased dose of PDN	Increased dose of PDN	Increased dose of PDN plus IVIG
Time from cessation of melatonin to improvement	5 weeks	–	6 weeks

*MG* myasthenia gravis, *anti-AchR* antibodies against the acetylcholine receptor, *PDN* prednisone, *MA* mycophenolic acid, *IVIG* intravenous immunoglobulins, *MGFA* Myasthenia Gravis Foundation of America Clinical Classification, *MGFA-PIS* Myasthenia Gravis Foundation of America Post-Intervention Status, *PR* Pharmacological Remission, *MM* Minimal Manifestations, *ADL* Activities of Daily Living Profile

## Case presentation

We identified three patients in our referral centre from 2014 to 2019 who presented a clinical exacerbation of myasthenia gravis after starting melatonin treatment.

The study was approved by the Ethics Committee of Bellvitge University Hospital (PR162/20).

### Case 1

The patient was a 49-year-old woman with generalized MG, negative for both anti-AchR and anti-MuSK antibodies and with no signs of thymoma on chest computed tomography (CT) scan. She was being treated with prednisone 10 mg/48 h and mycophenolic acid 360 mg/12 h, and had been asymptomatic for MG for over a year (ADL: 0, MGFA: 0, MG-PIS: PR (https://myasthenia.org/What-is-MG, Appendix in Table 2, Appendix in Table 3 and Appendix in Table 4). Due to persistent insomnia she began taking an over-the-counter melatonin supplement, 1.95 mg/day. A few weeks later she presented with constant binocular diplopia, dysphonia and generalized tiredness (ADL: 5), with no other identifiable triggers. Due to the temporal relationship between her symptoms and starting the melatonin supplement she was advised to cease taking it. She did not start treatment with pyridostigmine due to gastrointestinal inotolerance. As little improvement was initially observed in the next week, the dose of prednisone was increased to 50 mg/24 h. The patient subsequently showed a progressive improvement and recovered her baseline status at five weeks.

### Case 2

The patient was an 87-year-old man with generalized MG, positive for anti-AchR antibodies, without signs of thymoma on chest CT scan. The medical history included surgery for glottic carcinoma, with residual dysphagia. His MG symptoms remained stable for over six months with prednisone 15 mg/48 h (ADL 0, MGFA: I, MG-PIS: MM1), and subsequently the dose was reduced to 10 mg/48 h due to irritability. With this change the patient remained stable for six weeks. Due to difficulty falling asleep he began to take melatonin 1.9 mg each night. Two days later he presented constant ptosis during the day and increased dysphagia (ADL: 4) without evidence of glottic carcinoma recurrence. After returning to the initial dose of prednisone, plus pyridostigmine as needed, his symptoms stabilized but did not improve. He was advised to cease the melatonin, but preferred to continue taking it due to the positive effect on his insomnia.

### Case 3

The patient was a 35-year-old man diagnosed with generalized MG, positive for anti-AchR antibodies. Chest CT scan showed no thymoma, the patient having undergone a thymectomy (thymus hyperplasia). He was being treated with pyridostigmine 60 mg/4 h, mycophenolic acid 540 mg/day in two divided doses and prednisone 15 mg/48 h. With this treatment his MG symptoms were well controlled for over a year, and he only presented intermittent diplopia during the day (ADL: 2, MGFA I; MGFA-PIS: MM3). After a period of six months during which he suffered from insomnia, he began treatment with doxylamine, with no worsening of his MG, but this treatment was suspended due to ineffectiveness. One month after cessation of doxylamine, and due to the persistence of his insomnia, he began taking an over-the-counter supplement containing melatonin 1.95 mg/day. One week later he presented a progressive worsening of his MG, with no other identifiable trigger. Neurological examination revealed facial muscle weakness, difficulty chewing and appendicular weakness and fatigability (ADL: 7 points). He ceased taking melatonin, but in the absence of improvement over the next few days the dose of prednisone was increased to 30 mg/24 h. The patient’s ADL score remained at 6 for the following month and it was thus decided to administer intravenous immunoglobulins at a dose of 0.4 g/kg/day for 5 days. Two weeks after this treatment the patient experienced a marked clinical improvement, and at one month he had returned to his baseline status.

## Discussion and conclusions

Melatonin and its agonists are widely used in the treatment of insomnia, which has a prevalence in the general population of 30–50% [10, 11]. In some European countries, melatonin supplements may be purchased over the counter (without prescription) in pharmacies, provided that the tablets contain less than 2 mg of the active substance. Importantly, the use of melatonin among the general population has increased in recent years due to it being indicated as a treatment for REM sleep disorders [12], jet lag, chronic migraine, cluster headache [13] and chronic pain [14].

Another less well known function of melatonin is as an immune system regulator, it modulates both the innate and specific immune response. Here we describe three patients with MG whose symptoms were well controlled with a low maintenance dose of corticosteroids (plus, in two cases, with mycophenolic acid as a steroid-sparing agent) and who presented a worsening of their condition within days or weeks of starting melatonin. The fact that the exacerbation of MG symptoms manifested some days after starting melatonin suggests a mechanism of action other than its direct sedative effect, which would have occurred within an hour of taking it [12].

All patients were asked about recent infectious diseases, increased stress, surgery, changes in their usual treatments or other supplement intake. Although in Patient 1 there was no other identifiable trigger, possible triggers were present in the other two cases: a reduction in the maintenance dose of prednisone in Patient 2 and prescription of doxylamine in Patient 3. However, Patient 2 remained stable for six weeks with the reduced dose of glucocorticoid, while Patient 3 showed no change in symptoms in the month following cessation of doxylamine. By contrast, both patients presented neurological deterioration several days after beginning to take a melatonin supplement, hence the suspicion that melatonin had a role in their clinical exacerbation.

In none of the three patients did an increased dose of corticosteroids lead to meaningful clinical improvement in the next month. Patient 1 took five weeks to achieve symptom remission following cessation of melatonin, while Patient 3 was administered intravenous immunoglobulins four weeks after ceasing melatonin because his daily activities were still limited as a result of the symptom exacerbation. Patient 2 only experienced a worsening of his ocular symptoms and otherwise remained stable despite continuing to take the melatonin supplement. This may be because his MG symptoms were previously well controlled with a low dose of prednisone, whereas the other two patients (1 and 3) were also receiving an immunosuppressant, whose action was likely to have been affected by the introduction of melatonin.

The immunomodulatory effect of melatonin has become better understood in recent year. On the one hand melatonin seems to downregulate the overreaction of the innate immune response and promotes the adaptive immune activity. It enhance B lymphocytes production and stimulates T helper (Th) cell activity [6, 15] that play an important role in MG pathogenesis. On the other hand, it also increases the production of proinflammatory interleukin (IL). IL2 can induce resistance to glucocorticoids (both endogenous and exogenous) in Th cells [16] and may explain why these patients did not respond to an increased dose of glucocorticoids, when previously they had done. This effect is likely to persist beyond the plasma elimination [17] of exogenous melatonin, which would also account for why these patients did not improve until several weeks after cessation of melatonin.

The role of melatonin in autoimmune disorders such as rheumatoid arthritis and Crohn’s disease has previously been reported [15, 16, 18]. In the literature there are three case reports of autoimmune hepatitis after treatment with melatonin: two involved patients with no prior autoimmune disease [8, 9], while the other was a patient with primary sclerosing cholangitis and ulcerative colitis whose treatment included mycophenolate mofetil [7]. The three cases described here are the first in which melatonin administration was associated with symptom exacerbation in patients with MG who were being treated with immunosuppressants.

The use of melatonin in patients with MG, whether ocular or generalized, may trigger exacerbations of the disease, probably due to an upregulation of adaptative immune response and an interaction with treatment involving corticosteroids and other immunosuppressants. We consider that melatonin should be administered with caution in patients with MG, especially those who are being treated with immunosuppressants.

## Data Availability

The data that support the findings of this study are available from the corresponding author, [CC], upon reasonable request.

## Appendix 1

**Table 2:** Activities of Daily Living (ADL) Score range from 0 to 24 points. It was created to assess the impact of MG on activities of daily living. This scale includes eight functions that are often impaired in MG. It surveys symptom severity, with each response graded from 0 (normal) to 3 (most severe), (https://myasthenia.org/What-is-MG)

Grade	0	1	2	3
**Talking**	Normal	Intermittent slurring or nasal speech	Constant slurring or nasal, but can be understood	Difficult to understand speech
**Chewing**	Normal	Fatigue with solid food	Fatigue with soft food	Gastric tube
**Swallowing**	Normal	Rare episode of choking	Frequent choking necessitating changes in diet	Gastric tube
**Breathing**	Normal	Shortness of breath with exertion	Shortness of breath at rest	Ventilator dependence
**Impairment of ability to brush teeth or comb hair**	Normal	Extra effort, but no rest periods needed	Rest periods needed	Cannot do one of these functions
**Impairment of ability to arise from chair**	Normal	Mild, sometimes uses arms	Moderate, always uses arms	Severe, requires assistance
**Double vision**	Normal	Occurs, but not daily	Daily, but not constant	Constant
**Eyelid droop**	Normal	Occurs, but not daily	Daily, but not constant	Constant

## Appendix 2

**Table 3:** Myasthenia gravis foundation of America (MGFA) Clinical Classification. The sclae divides MG into 5 classes and several subclasses to identify subgroups of patients with MG who share distinct clinical features or severity of disease that may indicate different prognoses or responses to therapy, (https://myasthenia.org/What-is-MG)

**Class I:** Any ocular muscle weakness; may have weakness of eye closure. All other muscle strength is normal.	
**Class II:** Mild weakness affecting muscles other than ocular muscles; may also have ocular muscle weakness of any severity. **A. IIa.** Predominantly affecting limb, axial muscles, or both. May also have lesser involvement of oropharyngeal muscles. **B. IIb.** Predominantly affecting oropharyngeal, respiratory muscles, or both. May also have lesser or equal involvement of limb, axial muscles, or both.	
**Class III:** Moderate weakness affecting muscles other than ocular muscles; may also have ocular muscle weakness of any severity. **A. IIIa.** Predominantly affecting limb, axial muscles, or both. May also have lesser involvement of oropharyngeal muscles. **B. IIIb.** Predominantly affecting oropharyngeal, respiratory muscles, or both. May also have lesser or equal involvement of limb, axial muscles, or both.	
**Class IV:** Severe weakness affecting muscles other than ocular muscles; may also have ocular muscle weakness of any severity. **A. IVa.** Predominantly affecting limb, axial muscles, or both. May also have lesser involvement of oropharyngeal muscles. **B. IVb.** Predominantly affecting oropharyngeal, respiratory muscles, or both. May also have lesser or equal involvement of limb, axial muscles, or both.	
**Class V:** Defined as intubation, with or without mechanical ventilation, except when employed during routine postoperative management. The use of a feeding tube without intubation places the patient in class IVb.	

## Appendix 3

**Table 4:** MGFA Post-intervention Status (MGFA-PIS)

**Complete Stable Remission (CSR)** The patient has had no symptoms or signs of MG for at least 1 year and has received no therapy for MG during that time. Isolated weakness of eyelid closure is accepted.	
**Pharmacologic Remission (PR)** The same criteria as for CSR except that the patient continues to take some form of therapy for MG. Patients taking cholinesterase inhibitors are excluded from this category.	
**Minimal Manifestations (MM)** The patient has no symptoms of functional limitations from MG but has some weakness on examination of some muscles. **MM-0**: The patient has received no MG treatment for at least 1 year. **MM-1**: The patient continues to receive some form of immunosuppression but no cholinesterase inhibitors or other symptomatic therapy. **MM-2**: The patient has received only low-dose cholinesterase inhibitors (< 120 mg pyridostigmine/day) for at least 1 year. **MM-3**: The patient has received cholinesterase inhibitors or other symptomatic therapy and some form of immunosuppression during the past year.	

## Abbreviations

MG: Myasthenia gravis
anti-AchR: Acetylcholine receptor
anti-MuSK: Muscle-specific kinase
CT: Computed tomography
ADL: Activities of Daily Living Scale
MGFA: Myasthenia Gravis Foundation of America Scale
MG-PIS: MGFA Post-intervention Status
PR: Pharmacologic Remission
MM: Minimal Manifestations
IL: Interleukin
Th: T helper
